# Degradation Kinetics of Disulfide Cross-Linked Microgels: Real-Time Monitoring by Confocal Microscopy

**DOI:** 10.3390/gels9100782

**Published:** 2023-09-25

**Authors:** Iris G. Mercer, Angelina N. Italiano, Irina G. Gazaryan, Aaron B. Steiner, Sergey V. Kazakov

**Affiliations:** 1 Department of Chemistry and Physical Sciences, Pace University, Pleasantville, NY 10570, USA; iris.mercer@columbia.edu (I.G.M.); angelinaitaliano00@gmail.com (A.N.I.); igazaryan@pace.edu (I.G.G.); 2 Department of Biology, Pace University, Pleasantville, NY 10570, USA; asteiner@pace.edu

**Keywords:** redox-sensitive microgels, reducing agent, glutathione, kinetics of degradation, rate constants, confocal microscopy

## Abstract

Although biodegradable microgels represent a useful drug delivery system, questions remain regarding the kinetics of gel degradation and subsequent drug release. Spherical microgels (~Ø10–300 µm) were synthesized using an inverse suspension polymerization method. Specifically, acrylamide and acrylonitrile monomers were thermally co-polymerized with *N*,*N*’-bis(acryloyl)cystamine as a cross-linker with disulfide bridges. The kinetics and mechanism of degradation of these cross-linked, degradable, fluorescently labeled microgels (PAAm-AN-BAC-FA) were quantitatively studied under confocal microscopy at various concentrations of glutathione (reducing agent) ranging from 0.06 to 91.8 mM. It was found that polymer network degradation via the cleavage of disulfide bonds was accompanied by two overlapping processes: diffusion-driven swelling and dissolution-driven erosion. A slow increase in microgel size (swelling) resulted from partial de-cross-linking in the bulk of the microgel, whereas a faster decrease in fluorescence intensity (erosion) resulted from the complete cleavage of disulfide bonds and the release of uncleaved polymeric chains from the microgel immediate surface into the solution. Swelling and erosion exhibited distinct kinetics and characteristic times. Importantly, the dependence of kinetics on glutathione concentration for both swelling and erosion suggests that degradation would occur faster in cancer cells (higher concentration of reductants) than in normal cells (lower concentration of reductants), such that drug release profiles would be correspondingly different. A greater comprehension of microgel degradation kinetics would help in (i) predicting the drug release profiles for novel multifunctional drug delivery systems and (ii) using redox-sensitive degradable hydrogel particles to determine the concentrations of reducing agents either in vitro or in vivo.

## 1. Introduction

Spherical bipartite structures made of a hydrogel core enclosed within a lipid bilayer (“lipobeads”) may serve as nanometer sized, biocompatible, multifunctional systems for targeted anticancer drug delivery [1,2,3]. Being conceptually similar to lipid nanoparticles (LNP) [4,5], lipobeads could be promising for DNA/RNA therapeutics and for a wide range of infectious, genetic, neurodegenerative diseases and cancers. An ultimate goal in chemotherapy is superior tumor response and minimal side-effects even at high drug loading concentrations. The successful performance of lipobeads as versatile containers for targeted drug delivery and controlled drug release relies on the environmental responsiveness of the hydrogel core. Depending on the composition of the gel/solvent system, the polymer used, and cross-linking chemistry, the hydrogel core can swell or shrink discontinuously or continuously, reversibly or irreversibly, and in response to many different stimuli (temperature, pH, ion concentration, electric fields, light, reduction/oxidation, enzymatic activity, etc.) [6,7,8,9,10,11,12,13,14,15,16].

Keeping in mind the possible responses of a hydrogel core (contraction, swelling, and degradation) one could imagine three mechanisms of drug release from lipobeads, namely: “sponge-like”, a slow gradual drug release (hours/days); “poration”, a faster drug release (minutes/hours); and “burst”, “exploding” lipobeads with a drug release characteristic time of seconds. Three chemically different types of microgels supporting these three mechanisms have been synthesized in our research group [3]. So-called “thermophobic” poly(*N*-isopropylacrylamide-*co*-*N*,*N*’-methylenebis(acrylamide) bulk hydrogels and spherical microgels were shown to support the “sponge-like” mechanism, i.e., they shrink upon heating. The prepared “thermophilic” poly[acrylamide-acrylonitrile-*co*-*N,N*’-methylenebis(acrylamide)] bulk hydrogels and microgels exhibited the “poration” mechanism, i.e., they swell upon heating within the range of physiological temperatures. The synthesis and properties of degradable, spherical poly[acrylamide-acrylonitrile-*co*- *N,N*’-bis(acryloyl)cystamine] microgels suitable for the “burst” mechanism are the focus of this paper. The latter microgels comprise polymer chains cross-linked with disulfide bonds provided by *N,N*’-bis(acryloyl)cystamine (BAC). The disulfide-cross-linked polymer networks will degrade in the presence of reducing agents such as dithiothreitol (DTT), glutathione (GSH), N-acetylcysteine (NAC), etc. [13,14,15,16,17,18,19].

The reduced form of glutathione (GSH) was specifically chosen as a reducing agent in this work since this thiol-containing tripeptide is a major intracellular antioxidant [20]. In cancer cells, high levels of reduced glutathione and antioxidant systems are responsible for their increased survivability [21,22,23,24,25]. Active metabolism in cancer keeps reactive oxygen species (ROS) high enough for the constitutive expression of growth factors supporting proliferation, but insufficient to trigger apoptosis and ferroptosis. On the other hand, extreme concentrations of ROS result in widespread oxidative damage (stress) stimulating pro-death pathways, like it happens with the administration of many chemotherapeutics [22,24,25]. There is thus an interplay between the actual damage from ROS and ROS-induced protection through activation of the genetic antioxidant response, in particular Nrf2-driven expression of cytoprotective genes including those driving glutathione synthesis [26]. Continuous regeneration of GSH from the oxidized form of glutathione (GSSG) in the presence of glutathione reductase results in a prevalence of GSH over GSSG. The reverse conversion, catalyzed by glutathione peroxidase, reduces hydrogen peroxide to water, or lipid peroxides to lipid alcohols, the reaction directly implicated in ferroptosis. Glutathione acts as a redox buffer, and the $\left[GSH\right]:\left[GSSG\right]$ ratio serves as a metric of oxidative stress.

Although there are many difficulties associated with measuring the concentration of glutathione in cells (e.g., presence of gamma-glutamyltranspeptidase—the glutathione-degrading enzyme), higher glutathione concentrations in tumor tissue in comparison with normal tissue (with some exceptions, particularly in brain tissue) have been quite consistently reported [27,28,29,30]. For example, breast and lung cancer tissues contain anywhere from 1.5- to 10-fold the concentration of GSH found in normal cells. Measurements of glutathione in normal and cancer cells demonstrate that the total intracellular concentration of glutathione (GSH + GSSG) may vary from 0.03 to 3 mM in the normal cell, and from 1 to 100 mM in the cancer cell. As such, the highly reducing tumor environment allows for targeted drug release from biodegradable redox-sensitive delivery vehicles [31].

In this work, we report the synthesis of disulfide-cross-linked microgels and their degradation kinetics in the presence of glutathione as a reducing agent with naturally occurring concentrations ranging from 1 to 100 mM. Biodegradable polymeric systems are used in an increasing number of medical and pharmaceutical applications [15,16]. However, there are many interrelated factors affecting degradation behavior. A fundamental understanding of the polymer network degradation phenomena will allow the field to take full advantage of these materials, including the design and development of controlled drug delivery systems such as lipobeads. In the context of this work, degradation is considered as a chemical phenomenon (de-cross-linking of disulfide bonds), which results in complex physical processes such as swelling (diffusion-controlled) and surface and/or bulk erosion (dissolution-controlled), which can overlap and proceed simultaneously.

Degradation of the polymer network results from the overall reaction of disulfide cross-links reduction to thiols within the microgels, with concomitant oxidation of GSH into GSSG:

(1) $$\mathrm{RSSR}+2\mathrm{GSH}\overset{k}{\to }\mathrm{GSSG}+2\mathrm{RSH},$$

where RSSR is the microgel network cross-linked by disulfide bonds and RSH are the polymer chains separated after degradation. The rate law for this reaction can be represented by Equation (2):

(2) $$r=k{\left[\mathrm{GSH}\right]}^{X}{\left[\mathrm{RSSR}\right]}^{Y}$$

where k is the rate constant, $\left[\mathrm{GSH}\right]$ is the concentration of reduced glutathione, and $\left[\mathrm{RSSR}\right]$ is the concentration of the disulfide cross-links within microgels. This work aims to determine the partial orders X and Y and deduce the overall kinetics order (X+Y). We believe that better comprehension of the microgel degradation rate would help in predicting the drug release profile for lipobead-based drug delivery systems in the future.

The size of a realistic drug delivery system must be around 100 nm [32]; however, in this paper, we use microgels (~Ø10–300 µm) as a model permitting direct observation of their degradation behavior under optical fluorescence and confocal microscopes.

The complexity of degradation processes requires the use of advanced analytical techniques. Traditional non-optical [15], as well as fluorescence imaging [16], methods employed for measuring the material-mass loss in the course of polymer matrix degradation were able to follow only its erosion step. Standard optical (e.g., bright field) microscopy allowed one to observe only the swelling step in the degradation process [13,14]. Using confocal microscopy, we have developed a quantitative method for the real-time monitoring of all phenomena accompanying microgel degradation (swelling and erosion). For this purpose, spherical, fluorescently labelled, poly[acrylamide-*co*-acrylonitrile-*co*-bis(acryloyl)cystamine-*co*-fluoresceine-*o*-acrylate] microgels (PAAm-AN-BAC-FA) were synthesized. The observed integral fluorescence intensity (I_F_) was assigned to the concentration of disulfide bonds [RSSR] within an erodible microgel, whereas the measured diameter of the microgel (D) was assigned to the size of swellable microgel. The values of I_F_ and D were collected at multiple time intervals from the instant of glutathione injection. To the best of the authors’ knowledge, this paper is the first report on the quantitative separate characterization of microgel swelling and erosion observed simultaneously in the same degradation experiment. The kinetic parameters of microgel degradation at different concentrations of the reducing agent are calculated for swelling and erosion separately to infer the mechanism of degradation kinetics of disulfide cross-linked microgels.

## 2. Results and Discussion

### 2.1. Properties of Microgels

**Figure 1:**
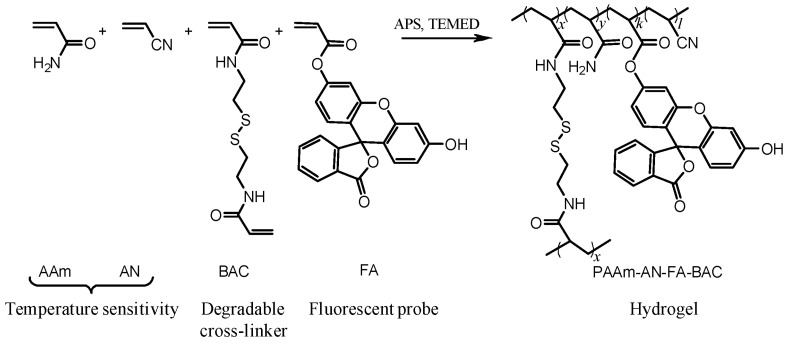
The polymerization scheme for preparation of PAAm-AN-BAC-FA microgels.

The synthesis of thermophilic, redox-responsive, fluorescent microgels was carried out in accordance with the polymerization scheme shown in Figure 1.

The relative amounts of all constituents of the hydrogel forming solution (HGFS) were optimized to achieve the desirable properties of the final product. To prepare thermophilic hydrogel, which expands/swells with temperature increase, a combination of acrylamide (AAm) and acrylonitrile (AN) was co-polymerized in a certain proportion. As previously reported [33,34], the temperature at which the volume phase transition of poly(acrylamide-*co*-acrylonitrile) occurs, T_V_, varies depending on the relative amounts of the monomers present. Based on these data, the AAm:AN molar ratio was kept at ~5.67:1 to obtain microgels that would be sensitive to temperatures within the physiological range.

**Figure 2:**
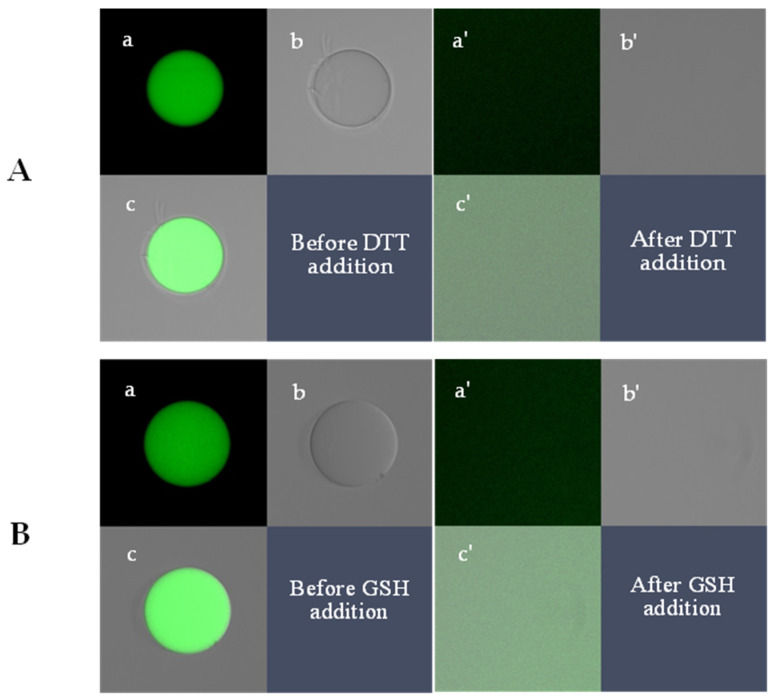
Confocal images of a PAAm-AN-FA-BAC microgel before (a,b,c) and after (a’,b’,c’) addition of DTT (**A**, 100 mM) and GSH (**B**, 126 mM) into a suspension of microgels in PBS buffer (pH 7.5): a,a’—green fluorescence images, b,b’—differential contrast images, c,c’—overlap images. All frames: 80 × 80 µm.

To optimize the extent of shrinking/swelling and minimize the interaction between fluorophore molecules (fluoresceine) covalently bound to the polymer chains, the Monomer:Cross linker:Fluorophore molar ratio was kept at ~20:1:0.0006. The microgels fluoresced as expected and were degradable in the presence of dithiothreitol (DTT) or GSH as the reducing agents (Figure 2). The immediate degradation of PAAm-AN-BAC-FA microgels was observed by confocal imaging when they were exposed to either 100 mM DTT buffer solution (Figure 2A) or 128 mM GSH buffer solution (Figure 2B) at pH 7.5. In both cases the microparticles completely disappeared under differential contrast microscopy (b,b’), indicating fast de-cross-linking of the microgel matrix, whereas fluorescence (a,a’) and overlay (c,c’) images (captured with equal exposure time and gain settings) exhibited a greenish background presumably resulting from the quick diffusion of the non-cross-linked fluorescently labelled polymer chains into the solution. The degradation was so fast that it was impossible to catch the intermediate states of degrading microgels; as such, further degradation experiments were carried out at lower concentrations of GSH ranging from 0.23 to 92 mM.

For the control experiment, spherical, fluorescently labelled, poly[acrylamide-*co*-acrylonitrile-*co*-methylene-bis-acrylamide-*co*-fluoresceine-*o*-acrylate] microgels (PAAm-AN-MBA-FA) were synthesized by the inverse suspension polymerization keeping the same Monomer:Cross linker:Fluorophore molar ratio as for PAAm-AN-BAC-FA. *N,N*’-methylene-bis-acrylamide (MBA) is not a redox sensitive cross-linker. When exposed to 100 mM of either DTT or GSH buffer solutions, PAAm-AN-MBA-FA microgels did not degrade, i.e., no change in fluorescence intensity and size was observed by confocal imaging up to 20 min after the reductant addition.

**Figure 3:**
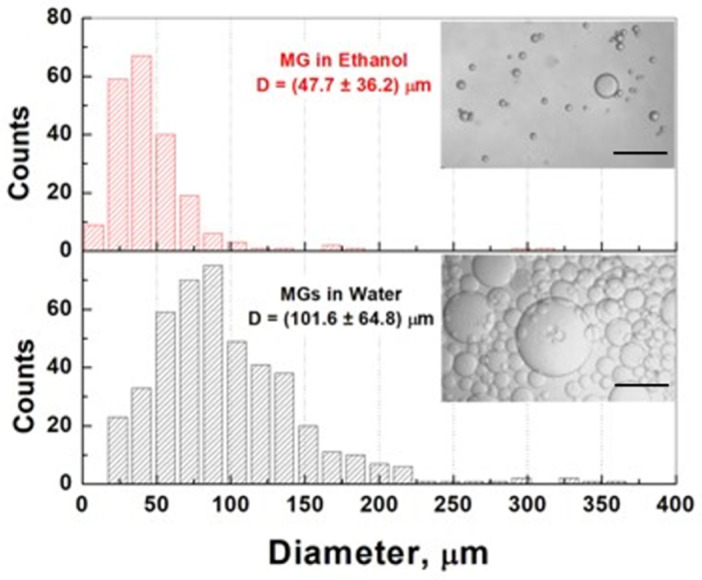
Size distribution of PAAm-AN-BAC-FA microgels in ethanol (shrunken) and in water (swollen). Scale bars: 200 µm.

PAAm-AN-BAC-FA microgel size distribution was examined in different solvents. Microgels in ethanol are opaque with diameters ranging from 0 to 100 μm, whereas in water they are transparent and range from 25 to 225 μm (Figure 3). Thus, PAAm-AN-BAC-FA microgels shrink in ethanol (similar behavior was found in other alcohols, not shown) and swell in water. The average volume-to-volume swelling ratio was calculated as $S_{V}=\frac{V_{W}}{V_{Et}}={\left(\frac{D_{W}}{D_{Et}}\right)}^{3}={\left(\frac{101.6}{47.7}\right)}^{3}\approx 9.7$. This finding was of high importance for the successful separation of microgels from cyclohexane (continuous phase) after inverse suspension polymerization, as well as for washing away unreacted components in the course of their preparation. The swelling/shrinking behavior of AAm-AN-based microgels in different solvents could have a wide range of applications, especially in medicine.

### 2.2. Confocal Imaging and Quantification of Microgel Size (D) and Fluorescence Intensity (I_F_)

**Figure 4:**
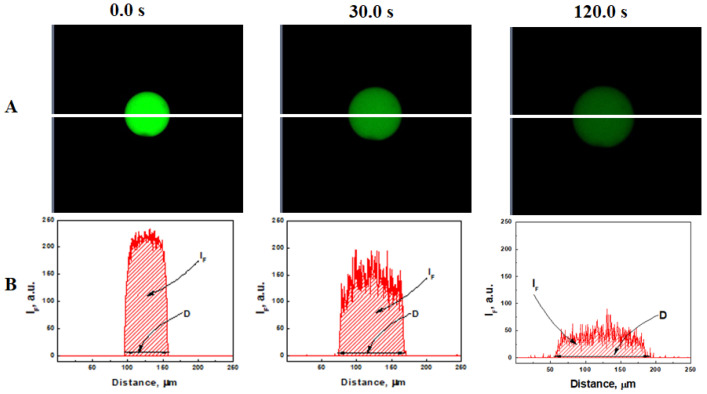
(**A**) Confocal fluorescence images of a PAAm-AN-BAC-FA microgel in PBS buffer (pH 7.5) before and after addition of GSH (2.3 mM). Green indicates fluorescein (FA, 488 nm excitation) covalently attached to the polymeric network. All frames: 256 × 256 µm. (**B**) Fluorescence intensity scanned along the white line in Panel A.

The redox degradation of prepared microgels was quantitatively studied in real time under confocal microscopy. Panel A in Figure 4 shows the microgel as a green area before (0 s) and after injection (30 s and 120 s) of 5 µL of 0.46 M glutathione PBS solution (pH 7.5) into the well containing microgels in 1 mL of PBS buffer solution (pH 7.5). The microgel clearly swelled (as a result of GSH diffusion into the bulk of the microgel and disulfide de-cross-linking), accompanied by a decrease in fluorescence intensity (presumably resulting from movement of uncleavable polymer chains outside the degrading microgel).

To quantify these observations, the fluorescence intensity was scanned along the white lines shown in Figure 4A. The integral fluorescence intensity ($I_{F}$) was calculated as the sum of all fluorescence signals along the scanning line, whereas the instant diameter of the microgel (D) was calculated as the distance between zero values of the fluorescence intensities on both sides of the particle (Figure 4B). The values of $I_{F}$ and D were collected at different time intervals from the instant of glutathione injection.

#### 2.2.1. Quantitative Analysis of Microgel Size Change in 2.3 mM GSH

**Figure 5:**
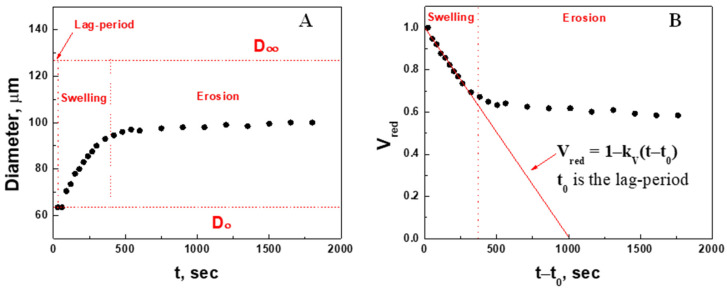
Time courses of diameter (**A**, original data) and reduced volume (**B**) of PAAm-AN-BAC-FA microgel in PBS buffer (pH 7.5) after addition of GSH (2.3 mM). Red line is the fitting of the pure swelling period to the function (4).

Microgel size changes over time in the presence of 2.3 mM GSH were recorded (Figure 5) using the above methodology. Three periods can be distinguished in the microgel size change: a lag period, swelling, and erosion. The lag-period ($t_{0}$) is a delay in swelling after GSH addition due to its diffusion into the microgel volume (bulk phenomenon). Erosion is a removal of polymer chains from the outermost layers of the microgel. Swelling is the result of de-cross-linking inside the microgel. Swelling kinetics should be observed between the initial and final diameters. The initial (D) diameter of the microgel was measured before GSH injection, whereas the final ($D_{\infty }$) diameter could not be determined experimentally because of the plateauing of diameter increase. The microgel swelling rate reduction may occur (i) at high concentrations of GSH, due to the erosion of its outermost layers or/and adhesion of the remaining layers of the microgel to the well bottom, and (ii) at low concentrations of GSH, due to the insufficient amount of the reductant (limiting reagent) to break all disulfide bonds. Whatever reason for the microgel slowdown in overall swelling, the final diameter $D_{\infty }$ can be found from fitting the data within the “pure swelling” period to the linear function in the straightening coordinates.

Since swelling is a three-dimensional phenomenon, its kinetics must be characterized in terms of volume changes, i.e., proportional to the cube of microgel diameter, $D^{3}$. The time-course of the changes in microgel volume was characterized by the reduced volume ($V_{red}$) calculated using Equation (3):

(3) $$V_{red}=\frac{D_{\infty }^{3}-D^{3}\left(t\right)}{D_{\infty }^{3}-D_{0}^{3}}$$

Only reduced data for “pure swelling” can be fitted into the linear function (4)

(4) $$V_{red}=1-k_{V}\left(t-t_{0}\right)$$

where $k_{V}$ is the rate constant of swelling, and $t_{0}$ is the lag period of swelling (Figure 5B). The linear dependence indicates that the swelling exhibits “zero”-order kinetics. We subsequently examined this phenomenon at other concentrations of GSH and compared the corresponding rate constant of swelling, $k_{V}$.

#### 2.2.2. Quantitative Analysis of Fluorescence Intensity in 2.3 mM GSH

**Figure 6:**
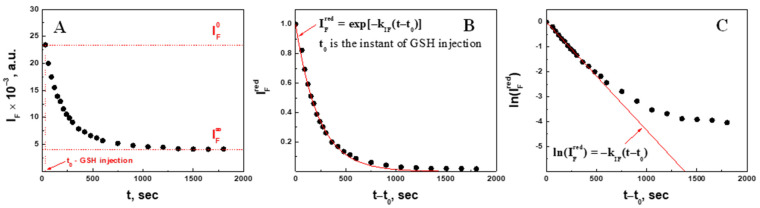
Time course of the original (**A**), reduced (**B**), and natural log of the reduced (**C**) fluorescence intensity of PAAm-AN-BAC-FA microgel in PBS buffer (pH 7.5) after addition of 2.3 mM GSH. Red line in panel (**C**) is the fitting to the function (6).

The fluorescence intensity decrease over time was quantified in the presence of 2.3 mM GSH (Figure 6A). The intensity changed between the initial intensity $I_{F}^{0}$ and final intensity $I_{F}^{*}$. Assuming statistical homogeneity in distribution of the fluorophore along the polymer chains within a degrading polymer network, it is reasonable to consider thereafter that $I_{F}\sim \left[\mathrm{RSSR}\right]$, meaning that the integral fluorescence intensity is proportional to the number of fluorophore molecules on the polymer chain. Therefore, a decrease in the fluorescence intensity can be assigned to the fluorescently labeled polymer chains released from the microgel into the solvent and their dissolution (cf. Figure 2) after breakage of the disulfide bonds according to the reaction (1). The movement of the fluorescently labeled polymer chains to the solvent begins from the microgel surface immediately after the addition of GSH, so that it was impossible to detect the lag-period, if any, by these methods and so that $t_{0}$ herein is the instant of GSH injection. “Pure decrease” in $I_{F}$ can be distinguished as long as there are enough molecules of the reductant to break all the S-S bonds along a polymer chain. It is anticipated that the higher the initial concentration of the reducing agent is, the lower the final intensity $I_{F}^{*}\;\mathrm{would}\;\mathrm{be}$, so that the ratio $(I_{F}^{0}-I_{F}^{*})/I_{F}^{0}$ can be considered as a measure of the extent of microgel degradation at different $\left[\mathrm{GSH}\right]$.

To compare the time changes in fluorescence intensity for the different concentrations of GSH on the same scale, the reduced intensity $I_{F}^{red}$ was calculated using Equation (5):

(5) $$I_{F}^{red}=\frac{I_{F}-I_{F}^{*}}{I_{F}^{0}-I_{F}^{*}}$$

Although $I_{F}^{red}$ is not a linear function of time (Figure 6B), $\ln\left(I_{F}^{red}\right)$ vs. time linearizes the portion of the dependence (Figure 6C), which can be ascribed to de-cross-linking accompanied by polymer chain dissolution (degradation). This portion of $\ln\left(I_{F}^{red}\right)$ data was fitted to the linear function (6):

(6) $$\ln\left(I_{F}^{red}\right)=-k_{\mathrm{IF}}\left(t-t_{0}\right)$$

where $k_{IF}$ is the rate constant of the fluorescence intensity decrease (degradation), and $t_{0}$ is the instant of glutathione injection. This analysis leads to the conclusion that reaction (1) is the first order with respect to RSSR, i.e., Y=1. The corresponding rate constants of degradation $k_{IF}$ for other concentrations of GSH were then compared.

### 2.3. Swelling Kinetic Parameters (Volume Change) of the Microgel at Different Concentrations of Glutathione

**Figure 7:**
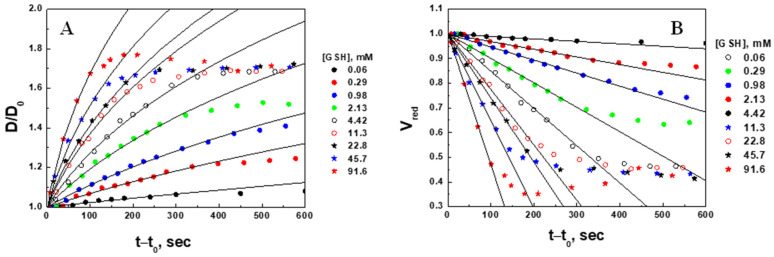
Time courses of the normalized diameter [**A**, solid curves calculated by (8)] and reduced volume [**B**, lines indicate fitting to function (4)] of PAAm-AN-BAC-FA microgel in PBS buffer (pH 7.5) after the addition of GSH.

Microgel swelling [normalized diameter (A) and reduced volume (B) change] were plotted in the same coordinates for all nine GSH concentrations studied (Figure 7).

Using the quantification analysis described in Section 2.2.1, the kinetic parameters resulting from the linear fitting procedure [initial ($D_{0}$) and final ($D_{\infty }$) diameters of the microgel, swelling ratio $S_{V}={\left(D_{\infty }/D_{0}\right)}^{3}$, apparent swelling rate constants (*k_V_*), swelling characteristic times ($\tau_{V}=1/k_{V}$), and regression correlation coefficient R] were recorded (Table 1).

Even though all the solutions were purged with N_2_ before each experiment to decrease the dissolved oxygen, the remaining concentration of dissolved oxygen was measured to be 1.4 mg/L. This means that a portion of injected GSH was consumed for the reduction of oxygen to water according to the well-known multistep process which can be described by the following overall reactions [35,36]:

(7) $$\left.\begin{array}{c} 2\mathrm{GSH}+O_{2}\to \mathrm{GSSG}+H_{2}O_{2}\\ 2\mathrm{GSH}+H_{2}O_{2}\to \mathrm{GSSG}+2H_{2}O \end{array}\right\}$$

As it follows from the reactions (7), the molar ratio of the consumed GSH to reduced O_2_ is 4:1. This has been confirmed experimentally as well [35]. Thus, 0.17 mM of GSH must be subtracted from the injected concentration (column 1) to correct the amount of GSH involved in the reduction of disulfide bonds. The corrected concentrations of GSH are presented in Table 1 (column 2) and in Figure 7.

It was mentioned in Section 2.2.1 that the initial ($D_{0}$) diameters of microgels were measured before GSH injection, whereas the final ($D_{\infty }$) diameters were found as fitting parameters. It is not surprising that the swelling ratios $S_{V}={\left(D_{\infty }/D_{0}\right)}^{3}\;\sim \;8$ are similar for all concentrations, since all the microgels studied were prepared in the same batch, so that they would statistically have the same cross-linking density.

The linear dependence of the reduced volume on time (Figure 7B) indicates that the swelling kinetics of microgels is “zero” order, i.e., diffusion-limited, given by the Peppas model [6,37], which proposes a power law $\Delta V\sim kt^{n}$ where the volume change is proportional to the time to the power of n. Herein, *n* is treated as an adjustable parameter, and the case of n=1 corresponds to so-called non-Fickian diffusion, whereas k depends on many structural and chemical factors of the gel, as well as on the concentration of the diffusing agent (in our case, GSH).

Rearranging Equations (3) and (4) with respect to the normalized microgel diameter $D/D_{0}$, the original data can be described by the nonlinear function (8) as shown in Figure 7A:

(8) $$\frac{D}{D_{0}}=\sqrt[{3}]{1+\left(S_{V}-1\right)k_{V}\left(t-t_{0}\right)}$$

In the range of GSH concentrations from 0.06 to 91.8 mM, the swelling characteristic times ($\tau_{V}=1/k_{V}$) vary from hours to a few minutes. The importance of this finding is that this range of GSH concentrations covers the physiological ranges of both normal and cancer cells.

Due to a visible concentration dependence of the zero-order swelling rate constant $k_{V}$, we call it the apparent rate constant. Therefore, the rate law for the swelling kinetics can be represented by Equation (9):

(9) $$r=k_{V}=k_{V}^{0}{\left[\mathrm{GSH}\right]}^{Z},$$

where $k_{V}^{0}$ is the true rate constant of swelling and Z is the swelling rate order with respect to GSH.

**Figure 8:**
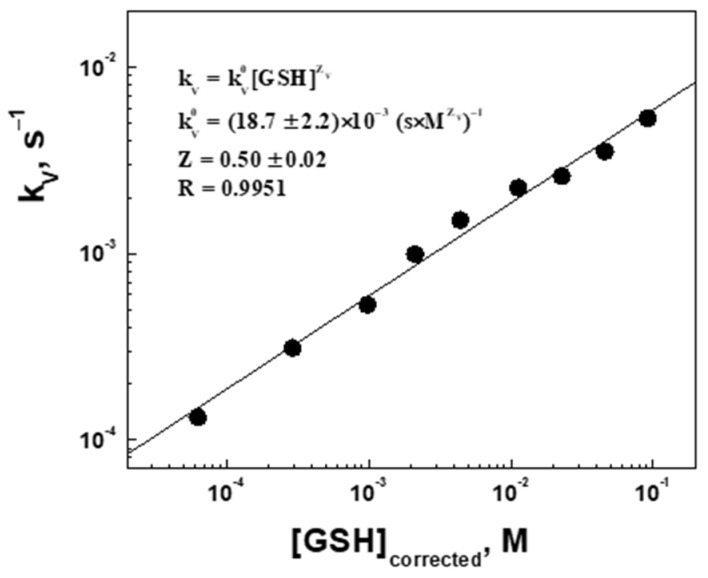
The apparent rate constant for the volume increase (swelling, $k_{V}$) as a function of the corrected glutathione concentration in the log-log coordinates.

To find $k_{V}^{0}$ and Z, the data on the apparent rate constant were analyzed in a double log straightening coordinate system, $log\left(k_{V}\right)$ vs. $log\left[\mathrm{GSH}\right]$ (Figure 8). The true swelling rate constant $k_{V}^{0}=\left(18.7\pm 2.2\right)\times 10^{-3}{\left(s\times M^{0.5}\right)}^{-1}$ is the same for all measured concentrations, whereas the half-order swelling kinetics (Z=0.50) with respect to GSH suggests that swelling will be faster in cancer cells (higher concentrations of reductants) than in normal cells (lower concentration of reductants), so that the drug release profiles will be correspondingly different. Furthermore, expression (9) can be used in the reverse direction, i.e., the cross-linked microgels can be used to determine the concentrations of reducing agents by measuring their swelling kinetics $\left[\mathrm{GSH}\right]\;\sim \;{\left(k_{V}\right)}^{2}]$, at least in vitro.

### 2.4. Degradation Kinetic Parameters (Fluorescence Intensity Decrease) of Microgel at Different Concentrations of Glutathione

**Figure 9:**
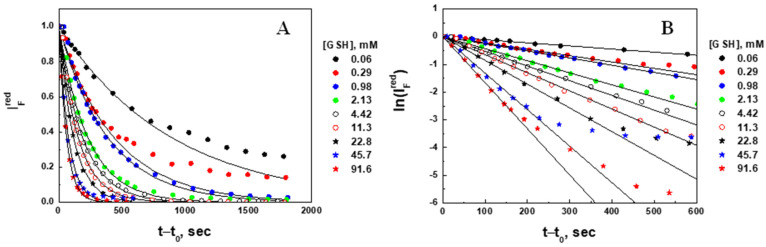
Time courses of the reduced fluorescence intensity (**A**) and natural logarithm of the reduced fluorescence intensity (**B**) of PAAm-AN-BAC-FA microgel in PBS buffer (pH 7.5) after the addition of GSH.

Microgel erosion [reduced fluorescence intensity (A) and natural logarithm of the reduced fluorescence intensity (B) change] was plotted in the same coordinates for all nine concentrations of glutathione studied (Figure 9).

Using the quantification analysis described in Section 2.2.2, the following kinetic parameters resulting from the linear fitting procedure are listed in Table 2: the initial ($I_{F}^{0}$) and final ($I_{F}^{*}$) fluorescence intensity, relative decrease in fluorescence intensity ($1-\frac{I_{F}^{*}}{I_{F}^{0}}$)—a measure of the extent of degradation (erosion) of the microgel, apparent erosion rate constants for fluorescence intensity ($k_{IF}$), characteristic times for erosion (fluorescence intensity decrease $\tau_{IF}=1/k_{IF}$), and regression correlation coefficient (R)—a measure of how well a linear regression model fits the $\ln\left(I_{F}^{red}\right)\mathrm{vs}.\;\left(t-t_{0}\right)$ data (Figure 9B). The concentrations of GSH were corrected the same way as in Section 2.2.1.

The initial ($I_{F}^{0}$) fluorescence intensities of the microgels were measured before GSH injection, whereas the final ($I_{F}^{*}$) intensities were found as fitting parameters. Since $I_{F}\sim \left[\mathrm{RSSR}\right]$, the decrease in the fluorescence intensity is proportional to the amount of uncleaved polymer chains leaving the microgel and dissolving in the surrounding solvent (erosion), so that the relative decrease in fluorescence intensity ($1-\frac{I_{F}^{*}}{I_{F}^{0}}$) can be considered as a measure of erosion or the extent of degradation of the microgel. Noticing that at {GSH] ≅ 92 mM the microgel was almost completely (93%) degraded, extrapolating the data to 100% degradation, and considering the molar ratio [RSSR]/[[GSH] = 2 (see Equation (1)), we estimate that the concentration of disulfide bonds (cross-linkers) within the gel is between 50 and 70 mM. This estimate is in agreement with the concentration of BAC (60 mM) in the hydrogel forming solution (see Section 4.2).

The comparison of the erosion ($\tau_{IF}=1/k_{IF}$) and swelling ($\tau_{V}=1/k_{V}$) characteristic times shows that erosion is more than a 3-fold faster process. Due to a visible concentration dependence, the first order erosion rate constant *k_IF_* is also considered the apparent rate constant which can be represented by Equation (10):

(10) $$k_{IF}=k_{IF}^{0}{\left[\mathrm{GSH}\right]}^{X},$$

where $k_{IF}^{0}$ is the true rate constant of erosion and X is the erosion rate order with respect to GSH.

**Figure 10:**
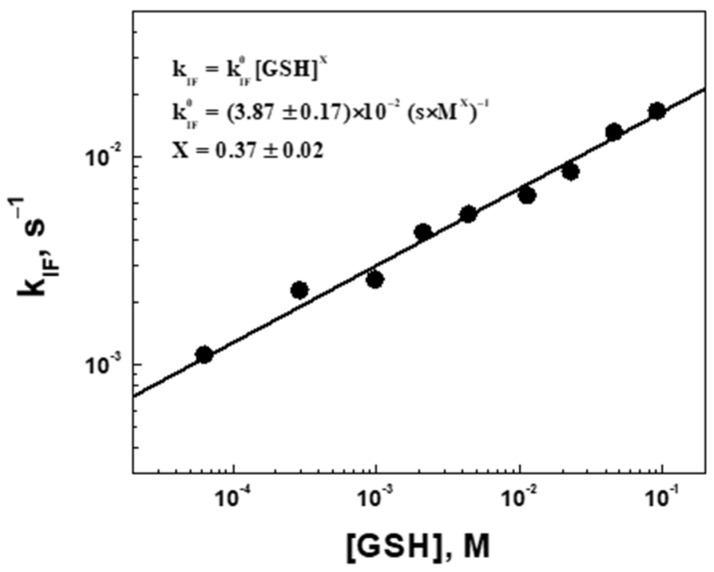
The apparent rate constant for a fluorescence intensity decrease (erosion, $k_{IF}$) as a function of the corrected glutathione concentration in the log-log coordinates.

To find $k_{IF}^{0}$ and X, the apparent rate constant was analyzed in double log straightening coordinates, $log\left(k_{IF}\right)$ vs. $log\left[\mathrm{GSH}\right]$ (Figure 10). The true erosion rate constant $k_{V}^{0}=\left(38.7\pm 1.7\right)\times 10^{-3}{\left(s\times M^{0.37}\right)}^{-1}$ is the same for all measured concentrations. The order of erosion kinetics is X=0.37 with respect to GSH, suggesting that degradation would be faster in cancer cells in comparison with normal cells. The overall rate law for the erosion kinetics can be represented by Equation (11):

(11) $$r=k_{IF}^{0}{\left[\mathrm{GSH}\right]}^{0.37}\left[\mathrm{RSSR}\right],$$

with the partial order X=0.37 in GSH, the partial order Y=1 in RSSR and the overall order X+Y=1.37.

### 2.5. Mechanism of Degradation (Swelling and Erosion) of PAAm-AN-BAC-FA Microgel in PBS Buffer (pH 7.5) in the Presense of Glutathione as a Reducing Agent

**Figure 11:**
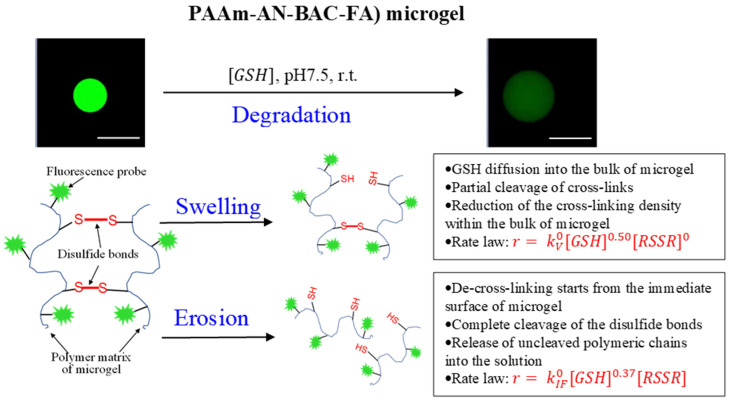
Proposed mechanism for degradation of disulfide cross-linked microgels after the addition of glutathione as a reducing agent. Scale bars: 100 µm.

The simultaneous quantitative measurements of microgel diameter and fluorescence intensity using confocal imaging allowed us to distinguish between two processes accompanying microgel degradation in the presence of different concentrations of the reducing agent (glutathione). The observed fluorescence intensity decrease and size of the microgel increase over the course of degradation can be explained by the following mechanism (Figure 11). Swelling is a slow 3D-process. It requires diffusion of GSH molecules into the bulk of a microgel causing a visible delay in swelling (lag-period) with respect to the instant of GSH injection. Partial cleavage of disulfide bonds is enough to reduce the cross-linking density within the bulk of a microgel and cause its swelling. Thus, it is reasonable that the slow diffusion-driven swelling exhibits kinetics independent of the concentrations of cross-links $\left[\mathrm{RSSR}\right]$ (zero-order] and depends on the initial gradient of GSH concentration (half-order kinetics).

On the contrary, the complete cleavage of disulfide bonds within the outermost layers starts immediately after GSH injection, causing a release of uncleaved polymer chains into the surrounding solution. This erosion process is much faster and depends not only on the concentration of glutathione (0.37-order kinetics), but also on the concentration of cross-links. Thus, first-order kinetics with respect to $\left[\mathrm{RSSR}\right]$ was observed.

## 3. Conclusions

In this study we have described a way to apply confocal microscopy for the simultaneous quantitative characterization of microgel swelling and erosion accompanying the microgel degradation process in the presence of different concentrations of a reducing agent.

Novel disulfide cross-linked, fluorescently labelled, spherical poly[acrylamide-*co*-acrylonitrile-*co*-bis(acryloyl)cystamine-*co*-fluoresceine-*o*-acrylate] microgels (PAAm-AN-BAC-FA, Ø10–300 µm) were synthesized using inverse suspension polymerization. These exhibit a thermophilic behavior and contraction/swelling in different solvents and display chemical sensitivity causing degradation in the presence of reducing agents as a result of disulfide bond de-cross-linking.

Following the addition of the reduced form of glutathione, redox degradation of these microgels was quantitatively studied in real time using confocal microscopy. The values of the integral fluorescence intensity and the diameter of the microgel were for the first time simultaneously measured at different time intervals from the instant of glutathione injection. The quantitative analysis of integral fluorescence intensity and microgel size change in nine different concentrations of glutathione allowed us to calculate the kinetic parameters for microgel swelling and erosion and to deduce the mechanisms driving the degradation kinetics of disulfide cross-linked microgels.

The derived reaction orders for swelling and erosion predict that the degradation of microgels loaded with drugs will be different in cancer cells as compared with normal cells; we anticipate that degradation and therefore drug release will be enhanced in the cancer cell microenvironment based on elevated levels of glutathione.

Knowing the difference between the kinetics of two processes constituting the redox-sensitive degradation and keeping in mind that the physicochemical properties of the loaded drug may have a strong effect on drug release kinetics, the behavior of microgels loaded with drug remains to be determined. Also of interest are the different kinetics of “naked” microgels versus those covered with a lipid bilayer (lipobeads). Piecing together the physicochemical characteristics of microgels and eventually nanogels and lipobeads will lay the groundwork for the design and engineering of novel “smart” multifunctional delivery systems with predictable drug release profiles.

## 4. Materials and Methods

### 4.1. Chemicals and Materials

A 99% acrylamide (AAm, Sigma-Aldrich, St. Louis, MO, USA) and 99% acrylonitrile (AN, Sigma-Aldrich, St. Louis, MO, USA) as comonomers were used for the preparation of thermophilic hydrogels. A 98% *N,N*’-bis(acryloyl)cystamine (BAC, Fisher Scientific, Hampton, NH, USA) was used as a cross-linker. Fluorescein-*o*-acrylate (FA, λ_Ex_/λ_Em_ = 490/520 nm, Sigma-Aldrich, St. Louis, MO, USA) was used as a fluorescent monomer dissolved in methanol (4.9 mg/mL). Ammonium persulfate (APS, Fisher Scientific, Hampton, NH, USA) and tetramethylethylenediamine (TEMED, Acros Organics, Geel, ANTWERP, Belgium) were utilized as an initiator and an accelerator for thermal radical polymerization, respectively. Sorbitane monostearate (Span 60), as a surfactant, and solvents (cyclohexane, chloroform, methanol, and ethanol of chemical grade) were purchased from Sigma Aldrich (St. Louis, MO, USA). Water purified by RiOs-16 Essential Water Purification System (EMD Millipore, Burlington, MA, USA) at the resistivity of 16 MW × cm was used in all experiments. After a 30 min N_2_-purging through PBS stock solution (pH 7.5, Sigma Aldrich, St. Louis, MO, USA) the dissolved oxygen was measured (ODO electrode, Vernier, Beaverton, OR, USA) at the level of 1.4 mg/L. A stock solution of the reducing agent L-glutathione (Alfa-Aesar, Haverhill, MA, YSA) was made in phosphate buffer (PBS, pH 7.5) at 459 mM. All chemicals were used as purchased without further purification.

### 4.2. Microgel Synthesis

The disulfide cross-linked poly(acylamide-*co*-aclylonitrile-*co*-*N,N*’-bis(acryloyl)cystamine-*co*-fluorescein-*o*-acrylate) (PAAm-AN-BAC-FA) microgels with fluorescence ability were prepared using an inverse suspension polymerization (ISP) method described elsewhere [3]. In brief, the oil phase consisted of cyclohexane (40 mL) and Span 60 (11.6 mM) as an oil soluble surfactant was sonicated for 10 min and purged with nitrogen for 15 min in a 250 mL two-neck round-bottom flask under stirring at 600 rpm rate. Using 4-mL of the hydrogel forming solution (HGFS), the aqueous phase containing AAm and AN monomers (1200 mM in ratio 6:1), BAC cross-linker (60 mM), FA fluorescent monomer (37 µM), and APS initiator (27 mM), was injected dropwise into the oil phase (cyclohexane) under continuous 800 rpm–stirring and N_2_–purging. After 30 min of stirring, the polymerization was accelerated by adding TEMED (4.5 mM). After 10 min, the stirring was stopped; the suspension was placed in a water bath at 40 °C and left for 24 h to complete polymerization. The top layer of cyclohexane was removed, and the lower layer was dissolved in 40 mL of ethanol and transferred to a 50 mL centrifuge tube. After 2 h of soaking in ethanol, the suspension was centrifuged for 15 min at 4000 rpm (Durafuge 200, Precision, Winchester, VA, USA). After 3 times washing in ethanol, the procedure was repeated 3 times in water to ensure that all microgels dispersed in aqueous medium were free of unreacted chemicals. During the washing procedure, it was noticed that the microgels were in a shrunken state in ethanol, whereas in water they were in a swollen state. Further characterization of thus prepared microgels was performed microscopically (see Section 2.1. for details).

### 4.3. Instrumentation

#### 4.3.1. Optical Microscopy (OM) Imaging of Microgels

A National Optical Compound Phase Contrast Digital microscope (DC3-163-PH, Microscope World, Carlsbad, CA, USA) was used to observe and estimate the size, shape, and morphology of microgels. The microscope was equipped with a CCD camera with an integrated imaging system (Motic Image Plus 2.0 ML, Motic Instruments Inc., Richmond, BC, Canada). The samples for optical microscopy were prepared by placing an aliquot of a suspension (40 μL) between a 2-propanol cleaned depression glass slide and No. 1 coverslip. To prevent the evaporation of water during observation, the sample was sealed along the perimeter of the coverslip by a nail polish.

The diameters of all microgels on the optical images taken (ranged from 200 to 450 counts) were measured to determine the size distribution of microgels in different solvents. The calibration of optical images was performed using the size standard provided by the microscope manufacturer (Microscope World, Carlsbad, CA, USA).

The ability of thermophilic microgels to swell with temperature was tested microscopically using a temperature-controlled system (Bioscience Tools, San Diego, CA, USA), which included microscope stage (BTC-S), heating/cooling controller (BTC-100) and chamber for replaceable coverslips (CSC-22). A suspension of microgels was pipetted (10 μL) into a Ø9 mm well formed by the Grace Bio-Labs SecureSeal imaging spacer on a 22 mm × 22 mm coverslip. The other coverslip was pressed to prevent vaporization of the sample. The coverslip chamber with the sample was equilibrated at the desired temperature on the temperature-controlled stage for 5 min before optical images of microgels were taken at different magnifications. The temperature was controlled on the surface of the external coverslip within ±0.1 °C of the set temperature. The diameters of microgels at different temperatures were measured by the same method described in the previous paragraph.

Microgel degradability was quickly and qualitatively evaluated under a low magnification microscope (×20–×40). A well of the 48-wells glass bottom plate (MatTek Corp., Ashland, MA, USA) was filled with a 1 mL suspension of microgels and the disappearance of microgels after the injection of glutathione was observed.

#### 4.3.2. Confocal Laser Scanning Microscopy (CLSM) Imaging of Microgels

A confocal laser scanning microscope (LSM 700, Carl Zeiss Inc., White Plains, NY, USA) was used to image the fluorescently labeled microgels. Fluorescein-o-acrylate (λ_Ex_/λ_Em_ = 490/520 nm) covalently attached to the PAAm-AN network served as the fluorophore for all experiments. The intensity of the 488 nm laser beam used for excitation was kept constant for all the measurements.

For the measurements of degradation under the confocal microscope, the suspension of microgels was prepared by replacing water with a PBS buffer. Since the microgels precipitate in aqueous medium, the replacement could be performed without centrifugation by removing the supernatant above the microgels settled on the bottom of a vial and pouring in buffer solution. After 5 cycles of this procedure with 15 min intervals between the cycles, the suspension was left overnight to soak in the buffer and remove all residue of water inside the microgels. They were adjusted to a final pH of 7.5 by adding 0.05 M NaOH aqueous solution.

The measurements under the confocal microscope were carried out within one of the wells in a 48-well glass bottom plate (MatTek Corp., Ashland, MA, USA). To measure the degradation of microgels at fixed concentrations of glutathione, 50 µL of the microgel suspension, necessary volumes of PBS buffer, and stock solution of GSH were sequentially added into the well to keep the total volume of the sample constant (1 mL) for all concentrations of GSH.

A single microgel was first located in the bright field mode of the optical microscope at ×100, then in the acquisition mode the focus, scan speed, gain coefficient, and digital magnification were adjusted to keep the intensity of green fluorescence within the dynamic range (without signal saturation) inside the 256 µm × 256 µm frame. The total frame scan took 1.56 s.

To overcome photobleaching, which can compromise the fluorescence image quality, the time exposure to light sources was reduced by decreasing the frequency of excitation-emission cycles. It was experimentally found that a 30 sec-interval between the cycles provided a negligible loss of fluorescence by fluorescein during ~2000 s of total measurement time.

In the time-series mode, 20 to 60 images of a single microgel were collected every 30 s after the injection of the corresponding volume of the reducing agent stock solution at a given concentration. The collected data were saved as a video (.avi) file for demonstration, whereas the separate frames were analyzed offline using ZEN software (Carl Zeiss Inc., White Plains, NY, USA) to measure the integrated fluorescence intensity ($I_{F}$) and diameter (D) of the microgel before and at different instants after injection as described in Section 2.2.

### 4.4. Statistical Analysis

For a distinct GSH concentration, all data points ($D,\;I_{F}$) were taken for the same, but changeable, sample (microgel) at different instants during the same experiment. The statistics come from the fitting procedure giving the standard deviation (SD) for the fitting parameters and the value of the regression correlation coefficient (R), i.e., how close it is to 1. Based on the three direct measurements of D and $I_{F}$ for the different microgels at the same $\left[\mathrm{GSH}\right]$, we found that the reproducibility of the rate constants ($k_{V},\;k_{IF}$) deduced from the fitting procedure was within the SDs reported in the tables. The SD for the characteristic time was calculated as the propagation of errors.

## Figures and Tables

**Table 1:** Swelling ratios and apparent rate constants of the microgels for the different concentrations of glutathione added (results of linear regression of the reduced microgel volume, $V_{red}$).

$\left[\mathrm{GSH}\right]$, mM Injected	$\left[\mathrm{GSH}\right]$, mM Corrected	$D_{0}$, µm	$D_{\infty }$, µm	Swelling Ratio, $S_{V}$	Apparent Swelling Rate Constant, $k_{V}$, × 10^−4^ s^−1^	Swelling Characteristic Time, $\tau_{V}$, min	Regression Correlation Coefficient, R
0.230	0.063	83.2	166	7.9	1.32 ± 0.11	126 ± 11	0.9868
0.459	0.293	98.1	197	8.1	3.11 ± 0.12	53.6 ± 2.1	0.9923
1.15	0.984	91.5	183	8.0	5.29 ± 0.14	31.5 ± 0.8	0.9965
2.30	2.13	63.5	126	7.8	9.91 ± 0.31	16.8 ± 0.5	0.9957
4.59	4.42	115.8	232	8.0	15.1 ± 0.5	11.0 ± 0.4	0.9958
11.5	11.3	111.7	224	8.1	22.5 ± 1.2	7.41 ± 0.40	0.9914
23.0	22.8	100.1	199	7.9	26.0 ± 0.6	6.41 ± 0.15	0.9977
45.9	45.7	74.6	148	7.8	35.3 ± 0.9	4.73 ± 0.12	0.9954
91.8	91.6	80.0	160	8.0	53.0 ± 1.0	3.14 ± 0.06	0.9996

**Table 2:** Degradation ratios and apparent degradation rate constants of the microgels for different concentrations of glutathione added (results of linear regression of the reduced fluorescence intensity, $I_{F}^{reg}$).

$\left[\mathrm{GSH}\right]$, mM Injected	$\left[\mathrm{GSH}\right]$, mM Corrected	$I_{F}^{0}$, × 10^−3^ a.u.	$I_{F}^{*}$, × 10^−3^ a.u.	Degradation Ratio, $1-I_{F}^{*}/I_{F}^{0}$	Apparent Degradation Rate Constant, $k_{IF}$ × 10^−4^ s^−1^	Characteristic Time, $\tau_{IF}$, min	Regression Correlation Coefficient, R
0.230	0.063	31.3	16.0	0.49	11.2 ± 0.3	14.9 ± 0.4	0.9878
0.459	0.293	37.9	12.0	0.68	22.9 ± 0.7	7.28 ± 0.22	0.9968
1.15	0.984	34.3	7.3	0.79	25.8 ± 0.4	6.46 ± 0.10	0.9989
2.30	2.13	23.4	4.0	0.83	43.5 ± 1.1	3.83 ± 0.10	0.9977
4.59	4.42	44.5	6.9	0.84	53.0 ± 0.8	3.14 ± 0.05	0.9967
11.5	11.3	41.2	7.4	0.82	65.3 ± 0.9	2.55 ± 0.04	0.9990
23.0	22.8	13.5	1.7	0.87	85.1 ± 1.6	1.96 ± 0.04	0.9990
45.9	45.7	32.8	3.5	0.89	131.7 ± 4.8	1.27 ± 0.05	0.9960
91.8	91.6	28.7	2.0	0.93	166.2 ± 3.1	1.00 ± 0.02	0.9993

## Data Availability

Not applicable.
